# Seroprevalence of *Toxoplasma gondii *infection among horses in Tunisia

**DOI:** 10.1186/1756-3305-4-218

**Published:** 2011-11-22

**Authors:** Sonia Boughattas, Ramzi Bergaoui, Rym Essid, Karim Aoun, Aida Bouratbine

**Affiliations:** 1 Laboratoire de Recherche 05SP03, Laboratoire de Parasitologie, Institut Pasteur de Tunis, 13 Place Pasteur BP74, 1002 Tunis Belvédères, Tunisia; 2 Laboratoire de virologie, Institut de la Recherche Vétérinaire de Tunisie (IRVT), 20 Rue Djebel Lakdhar la Rabta 1006 Tunisia

**Keywords:** Toxoplasma, horse, antibodies, seroprevalence, MAT, Tunisia

## Abstract

**Background:**

The present study was conducted to investigate the serological survey of *Toxoplasma *antibodies in local.horses from three major regions: a neighbourhood of a city in the North (Sidi Thabet), a neighbourhood of a city on the coast (Monastir) and a neighbourhood of a city in the middle (Battan) of Tunisia (North of Africa).

**Methods:**

A total of 158 serum samples were obtained from clinically healthy horses which consisted of 111 (32 female, 79 male) 2-10 years old and 47 (11 female, 36 male) older than 10 years. All of the horses were tested for antibodies to *T. gondii *using the Modified Agglutination Test (MAT).

**Results:**

According to MAT results, antibodies to *T. gondii *were found in 28 (17.7%) of 158 sera with the titers of 1:20 in 20 horses, 1:40 in 1 horse, 1:80 in 2 horses, 1:160 in 2 horses, 1:320 in 1 horse and ≥1:640 in 2 horses. Anti-*T. gondii *antibodies were found in 18 (16.2%) of 111 horses (2-10 years old) and 10 (21.2%) of 47 horses (older than 10 years old). Six (13.9%) out of 43 female had anti-toxoplasma antibodies and 22 (19.1%) from 115 males remained positive.

**Conclusion:**

Statistically significant differences in age groups and genders were observed between the seropositive and seronegative horses using the Chi square X(2) test. Other statistical correlation was also reported concerning horse breed.

## Background

Toxoplasmosis is a globally distributed zoonosis with a clinical impact in the unborn fetus and in the immunosuppressed individual. Consumption of undercooked meat has been well established as a major risk factor for human *Toxoplasma gondii *infection worldwide. All warm blood animals are receptive to the parasite but with different rates between the species [1]. With few exceptions horses are considered one of the less sensitive species to the pathogenic effect of *Toxoplasma gondii *[2], however, when infected the parasite is considered to be associated with encephalomyelitis in horses. Moreover, traditionally in Tunisia, the undercooked meat of horse is recommended for pregnant women leading thus to infection of mothers [3]. No data are available on the prevalence of the parasite in horses in our country that is why we investigated the determination of its serological survey with different correlations between the geographic location, the gender, the age and the breed of the animal.

## Materials and methods

### Animal data

Blood samples were obtained from 158 horses from different farms in 3 districts: in the north (Sidi Thabet City), the coast (Monastir City) and the middle (Battan City) of Tunisia (North of Africa). Horses were fed in-house with no free grazing. Different breeds of horse were tested: Arabian horse (n = 61), Thoroughbred (n = 3), Barb (n = 31), Arab Pur Sang (n = 24), Breton (n = 2), Pony (n = 9), Arab/Barb (n = 24), Mogod (n = 2), Barb/Breton (n = 2). The different ages of animals were pooled into two groups: one group with an age below 10 years old (n = 111) and the second group with horses older than 10 years old (n = 47). 43 females and 115 males were screened.

### Blood sampling and serological examination

Blood samples were obtained via a jugular vein, as approved by the National consultative ethnical committee for life sciences and health, centrifuged at 2500 rpm for 10 min and sera were stored at -20°C until use. Antibodies to *T. gondii *were determined using an in house Modified Agglutination Test (MAT). Briefly, *Toxoplamsa *antigen was made by growing the parasite in mice intra-peritonealy followed by treatment with trypsin and fixation with formaldehyde. The whole antigen was used to coat 96 well U bottomed polystyrene plates. The sera were screened first at two dilutions 1:20 and 1:200 in 2-Mercaptoethanol/PBS buffer. The plates were shaken for 1 min and then covered and incubated at room temperature for at least 5 hours free of any vibrations. The test was considered positive when a layer of agglutinated antigen/serum was formed covering at least 50% of the bottom of the wells at one dilution at least. In negative wells, antigen precipitation is observed. The positive samples were then titrated by two fold dilution.

### Statistical analysis

Differences in the seroprevalence of *T. gondii *infected horses between the different regions, male and female, and different age groups were analyzed using a Chi square test calculated with Excel 2007 (Microsoft^®^). The *P *value < 0.05 was considered statistically significant. The correlation between the rates of infection in different horse breeds was analyzed by SPSS for windows v17 software.

## Results and discussion

Antibodies were found in 28 (17.7%) of the 158 horses with titers of 1:20 in 20 horses, 1:40 in 1 horse, 1:80 in 2 horses, 1:160 in 2 horses, 1:320 in 1 horse and ≥1:640 in 2 horses. Horses from the three cities were positive with higher prevalence in the Coastal City: Monastir 12 (30.7%) from 39 horses, followed by the Central City: Battan 15 (19.2%) from 78 horses. The City in North: Sidi Thabet, showed the lowest prevalence with 1 positive sample (2.4%) from 41 horses (Table 1). The statistical analysis showed that other factors (gender, age, and breed) reported in the present study affected prevalence of infection. The seroprevalence in the adult horses, (>10-year-old) 21.2%: 10 of 47, was significantly higher than those of young horses (≤ 10-years-old) 16.2%: 18 of 111. Seroprevalence in horses increased with age. In addition to this difference between the age groups, a difference in the subgroups of female and male horses was also significant. Indeed, the female horses had a significantly lower seroprevalence, (13.9%: 6 from 43) than the male, (19.1%: 22 from 115). The 28 positive horses consisted of 7 Arabian horses out of 61, 6 Arab/Barb breed out of 24, 5 Arab PS out of 24, 6 Barb out of 31, 1 Breton out of 2 and 3 Pony out of 9 horses tested. The statistical correlation analysis showed a high infection rate for the Arab breed (Table 2).

**Table 1:** Summary of the seroprevalence results

	**Sidi Thabet City**		**Monastir City**		**Battan City**	
	**Ni**	**N+**	**Ni**	**N+**	**Ni**	**N+**
	
Sex						
Male	14	0	28	7	73	15
Female	27	1	11	5	5	0
Total	41	1	39	12	78	15
Age						
2-10 years	31	0	38	11	42	7
> 10 years	10	1	1	1	36	8
Total	41	1	39	12	78	15

Ni: initial samples number, N+: positive samples number

**Table 2:** Data concerning the positives horse samples

Sample	City	Gender	Age (years old)	Breed	MAT titer
24	Sidi Thabet	Female	> 10	Arab	≥ 640
45	Monastir	Male	≤ 10	Arab	20
46	Monastir	Male	≤ 10	Arab	20
47	Monastir	Male	≤10	Arab	20
48	Monastir	Female	≤ 10	Arab	20
57	Monastir	Female	≤ 10	Arab PS	20
58	Monastir	Male	≤ 10	Arab PS	20
65	Monastir	Male	≤ 10	Arab PS	20
66	Monastir	Male	≤10	Arab PS	20
69	Monastir	Female	≤10	Barb	20
74	Monastir	Female	≤10	Barb	20
75	Monastir	Female	>10	Barb	20
80	Monastir	Male	≤10	Barb	80
80	Battan	Male	>10	Pony	80
84	Battan	Male	>10	Arab	320
89	Battan	Male	≤ 10	Arab	160
107	Battan	Male	≤ 10	Arab/Barb	20
108	Battan	Male	>10	Arab/Barb	20
112	Battan	Male	≤ 10	Arab/Barb	20
113	Battan	Male	≤ 10	Arab/Barb	20
114	Battan	Male	≤ 10	Arab/Barb	20
118	Battan	Male	>10	Arab/Barb	20
126	Battan	Male	≤10	Arab PS	40
137	Battan	Male	>10	Barb	≥ 640
142	Battan	Male	>10	Barb	20
149	Battan	Male	>10	Breton	160
151	Battan	Male	>10	Pony	20
155	Battan	Male	≤ 10	Pony	20

In the present study, the overall seroprevalence was 17.7%, which was far less than other reports from other countries in Africa. The serological survey of this parasite has previously been conducted only in two countries on the African continent: Nigeria and Egypt. In the first country, the overall rate of anti-toxoplasma antibodies, exceeds 30% among Polo horses, local breed and the Argentine breed [4]. In Egypt a first study reported an upper rate of 40% [5] but the sera samples were collected from horses with neurological clinical manifestations. Recently a prevalence rate of 25% has been reported in this country among draught horses [6].

Close prevalences are, however, noted in Argentina, 13.1% [7] and in Brazil 15.8% [8] diagnosed by the same serological test MAT. Lower survey results are mainly observed in Central Woyming with 0.36% [9], Suede with 0.5% [10] and in South Korea with 2.6% [11].

The present study showed that older horses (>10-year-old) were more likely to be seropositive than horses under 10-years-old, which provided further evidence for the increased risk of *T. gondii *infection with acquisition of age through longer contact with infective oocysts from the environment. It also suggests, that male horses are more sensitive to the infection by the parasite than female horses, which is the opposite to that reported in a previous study in Egypt [6]. Similar results were observed in Turkey [12] but in contrast to our work, their difference was not shown to be statistically significant.

For results on the animal breed, it is the first time that a correlation has been reported between toxoplasmic infection and different horse breeds. Arab horses seem to be the most sensitive breed for acquisition of the infection.

Concerning geographic location, the coastal city had the highest antibody prevalence in horses tested, more than the middle city and north one. It is known that coastal climatic conditions are more likely favorable to the infection survey. Moreover, cats could freely be present and the water supplied to the horses was not controlled. However, in the North city, very strict hygienic measures are applied in breed farming which could explain why this has the lowest prevalence. We tried to collect samples from representative locations in our country Tunisia. Southern cities were not included as horses are not frequently present in such areas. In addition, the south part is almost desert with climatic conditions not favorable at all to the survey of the parasite *Toxoplasma gondii*.

## Conclusion

In conclusion, except for the north city, toxoplasmic infection seems to be not rare in others cities. The difference could be, thus, associated with ecological/climatic conditions, type of farming, the presence of cats and the quality of water. The differences may be attributed, to, the breed differences in susceptibility to *T. gondii*. Moreover, the zoonotic potential of *T. gondii *in animals eaten as food is stressed, as in our traditions undercooked horse meat is recommended to pregnant women to have a healthier baby. These practices should be reviewed and special awareness, advice and recommendations should be made by physicians to the target population.

## Competing interests

The authors declare that they have no competing interests.

## Authors' contributions

SB carried out the antigen production, serological experimentations, statistical analysis and drafted the manuscript. RB sampled blood specimens from animals and was responsible for acquisition of data and their analysis. RS participated in statistical analysis. KA has been involved in revising the manuscript critically for important intellectual content. AB conceived the study and has given financial support. All authors approved the final version of the manuscript.
